# Sex differences in associations between hair glucocorticoids and internalizing symptoms in adolescents

**DOI:** 10.1016/j.cpnec.2025.100311

**Published:** 2025-07-08

**Authors:** Yasmine Zerroug, Marie-France Marin, Mara Brendgen, Miriam Beauchamp, Jean R. Séguin, Sylvana M. Côté, Catherine M. Herba

**Affiliations:** a Department of Psychology, Université du Québec à Montréal, Montreal, QC, Canada; b CHU Sainte-Justine Azrieli Research Center, Montreal, QC, Canada; c Research Center of the Institut Universitaire en Santé Mentale de Montréal, Montreal, QC, Canada; d Department of Psychiatry and Addictology, Université de Montréal, Montreal, QC, Canada; e Department of Psychology, Université de Montréal, Montreal, QC, Canada; f Department of Social and Preventive Medicine, Université de Montréal, Montréal, QC, Canada

**Keywords:** Adolescence, Sex differences, Hair cortisol concentrations, Hair cortisone concentrations, Cortisol/cortisone ratio, Internalizing symptoms

## Abstract

From adolescence onwards, internalizing symptoms, such as depressive and anxiety symptoms, are twice as prevalent in adolescent girls than boys. Dysregulation of the hypothalamic-pituitary-adrenal (HPA) axis, which controls production and regulation of glucocorticoids (cortisol and cortisone), is linked to depressive and anxiety symptoms. Findings on hair cortisol, cortisone and the cortisol/cortisone ratio in relation to these symptoms have been inconsistent, particularly in adolescent community samples. The ratio provides an indication of the active versus inactive balance of cortisol concentrations, as a proxy of 11-beta-hydroxysteroid dehydrogenase enzymes. In addition, few studies have investigated whether these associations are the same for adolescent girls and boys. Hair samples of 64 adolescent girls and 59 adolescent boys (aged between 14 and 15 years old) were analyzed using the liquid chromatography-mass spectrometry (LC-MS) extraction method. Internalizing symptoms were measured via validated self-reported online questionnaires. For adolescent boys, no associations between hair glucocorticoids and depressive or anxiety symptoms were found. For adolescent girls, the analyses revealed a positive association between hair cortisone concentrations and depressive symptoms. Our findings highlight significant sex differences in the mechanisms that might operate between glucocorticoid concentrations and internalizing symptoms. Future longitudinal studies could test the predictive, sex-dependent effect of hair glucocorticoids concentrations during adolescence on the development of internalizing disorders in adulthood. Gaining a deeper understanding of HPA axis functioning could help to identify youth who are at greater risk of developing stress-related psychopathologies.

## Highlights

• Adolescent girls show a link between hair cortisone and depressive symptoms.
• No association between hair glucocorticoids and symptoms in adolescent boys were found.
• Findings suggest sex-dependent mechanisms in glucocorticoids and internalizing symptoms.

## 1 Introduction

The prevalence of internalizing symptoms, such as depressive and anxiety symptoms, has been increasing among adolescents over the past decades [1,2]. Experiencing internalizing symptoms during adolescence increases the risk of developing psychopathology in adulthood [[3], [4], [5]]. Additionally, adolescent girls report more internalizing symptoms than adolescent boys, highlighting the onset of sex differences pertaining to mental health problems from adolescence onwards [2,[6], [7], [8], [9]]. Indeed, beginning in adolescence, women are twice as likely to develop internalizing disorders than men, a difference that is not observed during childhood [6,7]. During adolescence, brain regions and mechanisms underlying stress regulation reorganize due to hormonal and biological changes [2,3]. Thus, it has been hypothesized that adolescent girls and boys may have different physiological and emotional responses to stressors [2,7].

**Fig. 1:**
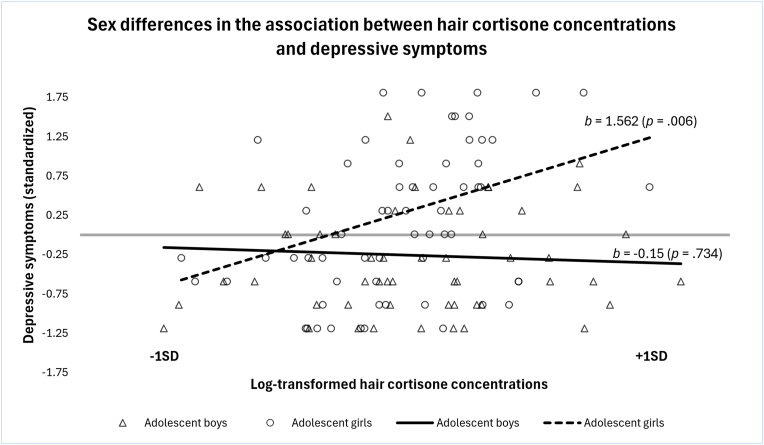
Post-hoc analysis showing a significant positive association between hair cortisone concentration and depressive symptoms in adolescent girls, whereas no significant association was found in adolescent boys.

The dysregulation of the hypothalamic-pituitary-adrenal (HPA) axis, controlling the production and regulation of glucocorticoids (cortisol and cortisone), has received attention as a potential physiological mechanism involved in the emergence and/or maintenance of internalizing disorders [[10], [11], [12], [13], [14]]. During stressful situations, a hormonal cascade is initiated, resulting in the release of cortisol, which plays a key role in the stress response by mobilizing energy to ensure survival. Cortisol is subsequently converted into cortisone, an inactive metabolite, by the enzyme 11-beta-hydroxysteroid dehydrogenase (11β-HSD) type 2 [10,13,15]. Cortisone can then be converted back into cortisol by 11β-HSD type 1. Once cortisol binds to neuroreceptors in the brain, a negative feedback loop is triggered to reduce hormone secretion and restore hormonal balance [10,13]. Cortisol and cortisone are interconvertible glucocorticoids that work together to help maintain physiological homeostasis. This dynamic conversion allows the body to regulate glucocorticoid activity in response to changing demands, particularly during perceived adversity. By modulating this balance, the system ensures an adequate distribution of energy resources to support adaptive responses to stress [16]. Adolescence is a period of significant brain plasticity that can be influenced by the maturation of the hypothalamic-pituitary-adrenal (HPA) axis during this stressful developmental phase. High concentrations of glucocorticoid receptors are found in the prefrontal cortex and limbic regions, where the regulation of cortisol and cortisone may play a crucial role. This regulation can impact the development of these brain areas and, consequently, influence the emergence of psychopathology [17].

While most previous research has quantified glucocorticoids with saliva samples, the use of hair samples is a different methodological approach that provides retrospective insight into cumulative glucocorticoid exposure over extended periods. This makes hair analysis particularly useful for research questions focusing on long-term HPA axis activity, whereas saliva may be more appropriate for capturing short-term or immediate fluctuations [[18], [19], [20]]. Disruptions in glucocorticoid regulation have been linked to internalizing symptoms and disorders in psychiatric clinical populations, yet findings have been mixed, and most studies have exclusively examined cortisol [11,12,14,[21], [22], [23]]. The findings across studies remain heterogeneous, with some reporting elevated hair cortisol concentrations in depressed and anxious individuals compared to control groups, while others observe reduced concentrations [12,24]. A meta-analysis of 16 original studies on hair cortisol concentrations in adults found that most studies did not report consistent or significant overall differences in cortisol concentrations between patients with major depression and control groups [25]. Another recent meta-analysis showed that compared to non-depressed controls, patients with major depression tended to have higher hair cortisol concentrations [26]. Despite the established importance of cortisol in stress-related disorders, research on hair cortisone and its relationship with internalizing symptoms is limited. Nonetheless, hair cortisone concentrations can provide valuable information on hormonal (dys)regulation by indicating how cortisol is metabolized (active versus inactive form), with the conversion between cortisol and cortisone, or vice versa, acting as an important biomarker of HPA axis functioning [14,15,20,[27], [28], [29]]. Further, evaluating the cortisol/cortisone ratio can deepen understanding of systemic glucocorticoid concentrations and potential issues with hormonal regulation, since the ratio acts as a proxy of enzymes 11β-HSD (type 1 and 2) activity [29,30]. Moreover, while examining these associations within a clinical population is valuable, it is equally crucial to investigate them in a community population, where internalizing symptoms are present on a continuum and below clinical threshold but nonetheless place adolescents at risk of developing internalizing disorders later in life [5,31].

Most community sample studies that have explored these associations between hair cortisol concentrations and depressive symptoms have yielded mixed results, with some observing positive associations [32,33] but others reporting negative [34] or no associations [23,35,36]. Interestingly, one study observed a curvilinear relationship between hair cortisol concentrations and depressive symptoms, where both low and high cortisol concentrations were observed in adolescents with high depressive symptoms, whereas those with moderate cortisol concentrations had low depressive symptoms [37]. Regarding anxiety symptoms, studies have reported negative associations with hair cortisol levels, with lower hair cortisol concentrations being associated with higher symptoms [32]. Another study found no significant links between hair cortisol and anxiety symptoms [35]. One study explored sex differences, although with a small sample size (n = 85), and found a positive and moderate association between hair cortisol concentrations and internalizing symptoms in adolescent boys but not in girls [32]. The same study also found a negative moderate association between hair cortisol concentrations and anxiety symptoms only in adolescent girls. Moreover, studies in community samples have not examined the role of hair cortisone concentrations and the few that did have focused on stress symptoms rather than internalizing symptoms [27,38]. Most studies have either overlooked sex differences or inadequately addressed them by simply controlling for sex in analyses. Thus, important information on how sex may moderate HPA axis functioning in relation to internalizing symptoms is lacking.

This study aimed to examine the associations between hair glucocorticoids and internalizing symptoms, and whether these associations were moderated by sex, in a community sample of adolescents. Based on the literature, we hypothesized that glucocorticoid concentrations and their ratio would be associated with depressive and anxiety symptoms and that sex would moderate these associations. For adolescent girls, we hypothesized that hair cortisol and hair cortisone concentrations would be positively related to depressive symptoms and negatively related to anxiety symptoms. For adolescent boys, associations with glucocorticoid concentrations and internalizing symptoms were exploratory. Given the heterogeneity of results in the literature, testing the contribution of the cortisol/cortisone ratio was also exploratory.

## 2 Material and methods

### 2.1 Participants and procedures

Participants were recruited from a longitudinal study [39,40], that followed families with children born between June 2003 and April 2004 (N = 497). The original participating families were recontacted via email to invite their study children to participate in a new follow-up study in adolescence [41], conducted in 2017 (T1, aged 13–14 years old) and 2018 (T2, aged 14–15 years old). Following parental consent and adolescent assent, 218 adolescents participated at T2 by completing an online questionnaire addressing various aspects of their social and emotional lives, in French or in English (95 % retention of T1, although significantly more adolescent boys dropped out after T1 than adolescent girls). Additionally, hair samples were collected from 126 adolescents (58 % of T2 participants) to assess glucocorticoids concentrations. These participants did not significantly differ from those who did not provide hair samples on key demographic or psychological variables. On average, hair samples were collected approximately one month after participants completed the online questionnaire. The present study sample consists of 126 adolescents, 65 girls and 61 boys, (M age = 14.08, SD = 0.27) from the T2 data collection. Of the 126 participants, one boy was excluded due to outlying cortisol concentrations values (10 standard deviation above the mean) and two participants (one boy, one girl) because data were missing for depressive and anxiety symptoms. A sample of 123 participants (59 boys, 64 girls) was used for analyses. This study was approved by the Research ethics board of the CHU Sainte-Justine Azrieli Research Center.

### 2.2 Measures

#### 2.2.1 Sample characteristics

Participants reported on sex (using the question: are you a boy or a girl?), age, anthropometric measures (i.e., weight, height) and substance use. Since no question on gender identity was asked, it was not possible to confirm that all participants were cisgender. Pubertal status was assessed using the Pubertal Development Scale, with a higher score indicating a more advanced pubertal development (PDS [42]). An in-house questionnaire was used to obtain information about hair treatment (e.g., dye, bleach, frequency of wash). Previous research has indicated that these variables could influence glucocorticoid concentrations, hence their consideration [14,36]. Parents self-reported on their adolescent's medication (i.e., is your adolescent taking any medication and if so, which one?) and the presence of any current diagnoses (i.e., has your adolescent been diagnosed with any physical or mental health condition and if so, which diagnoses?). Sample characteristics are presented in Table 1.

**Table 1:** Sample characteristics.

Characteristics	Total (n = 123)	Girls (n = 64)	Boys (n = 59)
Mean age in years (SD)	14.08 (0.27)	14.09 (2.90)	14.07 (0.25)
Use of prescribed medication			
Yes	17.89 % (*n = 22*)	17.18 % (*n = 11*)	18.64 % (*n = 11*)
No	82.11 % (*n = 101*)	82.82 % (*n = 53*)	81.36 % (*n = 48*)
Presence of self-reported mental health diagnosis			
Yes	26.83 % (*n = 33*)	28.13 % (*n = 18*)	25.40 % (*n =15*)
No	73.17 % (*n = 90*)	71.87 % (*n = 46*)	74.60 % (*n =44*)
Mean Child Depressive Inventory – Short Version (SD) ∗	1.4 (0.3)	**1.5 (0.4)**	**1.3 (0.3)**
Mean Revised Children's Manifest Anxiety Scale – Second Edition (SD) ∗∗	2.2 (0.6)	**2.4 (0.6)**	**1.9 (0.2)**
Mean Pubertal Development Scale (SD) ∗∗∗	N/A	**3.2 (0.5)**	**2.3 (0.6)**
Mean hair cortisol concentration (SD)∗∗∗∗	4.75 (6.16)	4.37 (4.31)	5.16 (7.74)
Mean hair cortisone concentration (SD)∗∗∗∗	11.32 (7.72)	10.47 (6.44)	12.26 (8.91)
Mean cortisol/cortisone ratio (SD)∗∗∗∗	0.47 (0.80)	0.40 (0.24)	0.55 (1.13)
Mean hair log cortisol (SD)	0.50 (0.36)	0.51 (0.31)	0.48 (0.41)
Mean hair log cortisone (SD)	0.97 (0.26)	0.96 (0.24)	0.99 (0.29)
Mean logcortisol/logcortisone ratio (SD)	0.47 (0.32)	0.50 (0.26)	0.45 (0.32)

Results that differed significantly between sex are in bold (p < .05). Glucocorticoid concentrations values are reported in pg/mg. ∗ Total score can range from 0 to 30. ∗∗ Total score can range from 0 to 57. ∗∗∗ Lower scores suggest earlier stages of puberty. ∗∗∗∗ Pre-transformed values.

#### 2.2.2 Child depression inventory short version (CDI-SV)

The CDI-SV [43,44] is a self-report questionnaire with ten items answered on a 3-point Likert scale, assessing the frequency of depressive symptoms. The mean of the ten items was used as the depressive symptoms score for analysis. The internal consistency within this sample was acceptable (Cronbach's α of .83).

#### 2.2.3 Revised Children's manifest anxiety scale – second edition short form (RCMAS-2)

The RCMAS-2 [43,45] is a self-report questionnaire with ten items answered on a 4-point Likert scale, assessing the frequency of anxiety symptoms. The mean of the ten items was also used as the anxiety symptoms score for analysis. The internal consistency within this sample was acceptable (Cronbach's α of .88).

#### 2.2.4 Hair samples

Hair samples were collected by participants at home. Participants were instructed to cut a sample of hair from the back of their head at the level of the ears and as close to the scalp as possible (posterior vertex position). All necessary materials were provided to participants, including detailed instructions and a link to a short video with additional information as well as surgical scissors. A stamped return envelope was provided to participants so they could mail the sample back in a freezer bag, as also done in other studies [46,47]. Glucocorticoid concentrations were extracted from the 3-cm of hair closest to the root. Studies have suggested that hair grows at an approximate rate of 1-cm per month, meaning that a 3-cm sample typically represents the accumulation of cortisol and cortisone over the preceding 3 months [20,36,48]. However, the accuracy of this estimate remains debated, as factors such as hair texture, type, and environmental influences can cause individual variations in hair growth rates, potentially affecting the representativeness of a 3-cm sample [49]. Hair samples were analyzed at Technische Universität Dresden in Germany, utilizing liquid chromatography-tandem mass spectrometry (LC-MS) for the extraction and measurement of glucocorticoid concentrations [48].

### 2.3 Statistical analyses

Analyses were conducted using IBM SPSS Statistics, version 29. To address positive skewness, cortisol and cortisone concentration values underwent a logarithmic transformation (LOG10). This transformation is commonly applied in endocrine research to correct for skewed data distributions [36]. LOG10 values were calculated after excluding one participant with highly improbable cortisol concentrations (10 standard deviation above the mean). The cortisol/cortisone ratio was calculated using log-transformed values and glucocorticoid concentrations were reported in pg/mg. Since certain variables are known to be associated with glucocorticoid concentrations [[14], [15],^36^], correlations (for continuous variables; age, body mass index (BMI), hair washing frequency, time in days between hair collection and sample analyses, pubertal status)) and t-tests (for binary/nominal variables; use of medication (yes or no), presence of a self-reported mental health diagnosis (yes or no), hair treatment practices (hair dye and bleach, coded as yes or no), hair collection season (spring, summer, fall)) were tested on glucocorticoid concentrations. Since fall was the only season where glucocorticoid concentrations significantly differed compared to the other seasons, we decided to merge spring and summer together (other). A significance p-value threshold of .10 was used to determine which variables should be included as covariates (conservative criterion utilized within our lab). Subsequently, only BMI, hair collection season (fall versus other) and hair treatment (dye/bleach versus no dye/bleach) demonstrated correlations with glucocorticoid concentrations and were thus included as covariates in analyses for cortisol, cortisone and the cortisol/cortisone ratio (see Table 2). To enhance statistical power through the reduction of variables in the models, initial regressions were performed with the covariates and each predictor variable (cortisol concentrations, cortisone concentration, and cortisol/cortisone ratio). The unstandardized residuals outcomes of these regressions were used as the new predictor variables of our main analyses to account for adjustment of these covariates. Since both symptom scores were not on the same scale, depressive scores and anxiety scores were standardized for comparison purposes. A linear model provided the best fit to the data and was therefore retained for all analyses.

**Table 2:** Potential covariates for hair glucocorticoids.

	Log cortisol concentrations (pg/mg)			Log cortisone concentrations (pg/mg)			logCortisol/logCortisone Ratio (pg/mg)		
*A. Bivariate correlations*		*r*	*p*		*r*	*p*		*r*	*p*
Age (in years)		.10	.268		.05	.56		.05	.53
**Body mass index (BMI)**		**0.19∗**	.041		.09	.345		**0.19∗**	.032
Hair washing frequency (per week)		−.02	.853		−.11	.239		.02	.835
Time between hair collection and analysis (days)		.07	.473		−.04	.644		.08	.36
Pubertal status		.07	.463		−.08	.392		.10	.265
*B. T-tests*	*N*	*t*	*p*	*N*	*t*	*p*	*N*	*t*	*p*
**Season of hair sample collection (fall vs. other)**	12	**−2.73∗**	.007	12	**−1.96∗**	.052	12	**−2.42∗**	.017
**Hair treatment (dye, bleach; yes/no)**	11	**−1.84∗**	.069	11	−1.48	.143	11	−1.58	.116
Use of medication (yes/no)	22	−1.03	.306	22	−0.35	.728	22	−0.94	.352
Presence of self-reported mental health diagnosis	33	−0.99	.325	33	−0.44	.66	33	−0.96	.341

Results in bold were included as covariates (unstandardized residuals) when calculating glucocorticoids concentrations. ∗ *p*-values <0.1.

To examine associations between glucocorticoid predictors and both depressive and anxiety symptoms, multivariate analysis of covariance (MANCOVAs) models were conducted. All assumptions to conduct MANCOVAs were met. Three MANCOVAs models were conducted: Model 1 with hair cortisol concentrations (unstandardized residuals) as the independent variable, Model 2 with hair cortisone concentration (unstandardized residuals) as the independent variable and Model 3 with cortisol/cortisone ratio (unstandardized residuals) as the independent variable. The same two dependent variables were examined in each model: depressive and anxiety symptoms. Sex (boy, girl) was added as a categorial variable in each model. To test sex differences, an interaction term between sex and glucocorticoid concentrations was also added in each model. MANCOVAs by sex were conducted to decompose the significant interaction and to assess the direction of sex differences. Moreover, pubertal status was added as a covariate in each model as it differed between sexes (Table 1) and was correlated with both dependent variables. Partial eta squared (partial η^2^) values (effect size) were used with the following benchmarks [50]: small (.01), medium (.06), large (.14).

Preliminary analyses showed that four participants reported using alcohol and cannabis more than once a week. As a test of sensitivity, we conducted main analyses with and without these participants to assess the impact on results. Since results remained unchanged in both cases, we decided to include these participants in our analyses to preserve statistical power.

## 3 Results

### 3.1 Sample characteristics and covariates for hair glucocorticoids

Sample characteristics are presented in Table 1. T-tests revealed that adolescent girls had higher scores then adolescent boys on the following variables: depressive symptoms, anxiety symptoms and the puberty score. There were small to medium positive correlations between puberty score and depressive (*r* = .22, *p* = .015) as well as anxiety symptoms (*r* = .26, *p* = .004). Potential covariates are presented in Table 2. There was a strong and positive association between hair cortisol concentrations and hair cortisone concentrations (*r* = .76, *p* < .001) and a medium positive correlation between depressive symptoms scores and anxiety symptoms scores (*r* = .52, *p* < .001).

### 3.2 Associations between hair glucocorticoids and internalizing symptoms

The results presented in this section represent the overall multivariate tests of each model, with detailed estimates and test statistics for individual variables reported in Table 3. Results for Model 1 revealed no significant main effect of cortisol concentration [*F*(2, 113) = 2.08, *p* = .13, η^2^ = .04], no significant main effect of pubertal status [*F*(2, 113) = 0.13, *p* = .881, η^2^ = .00] and no significant sex∗cortisol concentrations interaction [*F*(2, 113) = 2.46, *p* = .09, η^2^ = .04] on depressive and anxiety symptoms. A significant main effect of sex [*F*(2, 113) = 7.67, *p* < .001, η^2^ = .12] was found. Adolescent girls had higher levels of depressive and anxiety symptoms than adolescent boys. Model 2 showed no significant main effect of cortisone concentrations, [*F*(2, 113) = 1.94, *p* = .148, η^2^ = .03], no significant main effect of pubertal status [*F*(2, 113) = 0.20, *p* = .821, η^2^ = .00] and a significant main effect of sex [*F*(2, 113) = 7.64, *p* < .001, η^2^ = .12] on depressive and anxiety symptoms. Adolescent girls had higher levels of depressive and anxiety symptoms than adolescent boys. A trend for the interaction between sex and cortisone concentration emerged [*F*(2, 113) = 3.02, *p* = .053, η^2^ = .05]. A post-hoc analysis (see Fig. 1) indicated a positive association between cortisone concentration and depressive symptoms in girls (b = 1.56, *p* = .006) but not in boys. (b = −0.15, *p* = .734). No significant association was found for anxiety symptoms for either sex in model 2. Finally, results for Model 3 revealed no significant main effect of the cortisol/cortisone ratio [*F*(2, 113) = 1.76, *p* = .176, η^2^ = .03], no main effect of pubertal status [*F*(2, 113) = 0.15, *p* = .860, η^2^ = .00] and no significant sex∗ratio interaction [*F*(2, 113) = 1.42, *p* = .245, η^2^ = .03] on depressive nor anxiety symptoms. A significant main effect of sex was found [*F*(2, 113) = 7.03, *p* = .001, η^2^ = .11]. Adolescent girls had higher levels of depressive and anxiety symptoms than adolescent boys. Detailed results are presented in Table 3.

**Table 3:** MANCOVAs (3) – glucocorticoids and internalizing symptoms.

*Independent Variables*	*Dependent Variables*	*SS*	*df*	*MS*	*F*	*p*-value	Partial η^2^
(1) Cortisol concentration	Depressive symptoms	3.14	1	3.14	3.40	.068	.03
	Anxiety symptoms	2.18	1	2.18	2.67	.105	.02
Sex	Depressive symptoms	3.91	1	3.91	**4.25**^a^	.042	.04
	Anxiety symptoms	12.54	1	12.54	**15.37**^a^	<.001	.12
Puberty score	Depressive symptoms	0.13	1	0.13	0.14	.71	.01
	Anxiety symptoms	0.02	1	0.02	0.02	.892	.00
Sex∗Cortisol concentration	Depression symptoms	4.57	1	4.57	4.96	.028	.04
	Anxiety symptoms	0.98	1	0.98	1.21	.275	.01
(2) Cortisone concentration	Depressive symptoms	3.54	1	3.54	3.90	.051	0.03
	Anxiety symptoms	0.84	1	0.84	1.03	.312	0.01
Sex	Depressive symptoms	3.90	1	3.90	**4.29**^a^	.041	0.04
	Anxiety symptoms	12.48	1	12.48	**15.29**^a^	<.001	0.12
Puberty score	Depressive symptoms	0.28	1	0.28	0.31	.58	0.00
	Anxiety symptoms	0.00	1	0.00	0.00	.988	0.00
Sex∗Cortisone concentration	Depressive symptoms	5.23	1	5.23	**5.74**^a^	.018	0.05
	Anxiety symptoms	2.11	1	2.11	2.58	.111	0.02
(3) Cortisol/Cortisone ratio	Depressive symptoms	2.35	1	2.35	2.50	.117	0.02
	Anxiety symptoms	2.21	1	2.21	2.70	.103	0.02
Sex	Depressive symptoms	3.39	1	3.39	3.60	.060	0.03
	Anxiety symptoms	11.58	1	11.58	**14.15**^a^	<.001	0.11
Puberty score	Depressive symptoms	0.20	1	0.20	0.21	.644	0.00
	Anxiety symptoms	0.00	1	0.00	0.00	.957	0.00
Sex∗Cortisol/Cortisone ratio	Depressive symptoms	2.70	1	2.70	2.87	.093	0.03
	Anxiety symptoms	0.47	1	0.47	0.57	.452	0.01

a Significant results are in bold. Other significant *p*-values that are not in bold are not considered significant given that the main effect or the interaction term in the main model did not reach the significance threshold (see Results).

## 4 Discussion

This study aimed to test associations between hair glucocorticoid concentrations (cortisol and cortisone, cortisol/cortisone ratio) and internalizing symptoms (depressive and anxiety) for adolescent girls and boys. A significant positive association between hair cortisone concentrations and depressive symptoms was found for adolescent girls, but none of the glucocorticoid concentrations nor the cortisol/cortisone ratio were associated with anxiety symptoms. No significant associations were found for adolescent boys.

The hypothesis that hair glucocorticoid (cortisol and cortisone) concentrations and their ratio (cortisol/cortisone ratio) would be positively associated with depressive symptoms in girls was partially supported. While no significant associations were found between hair cortisol concentrations (nor the cortisol/cortisone ratio) and depressive symptoms, hair cortisone was associated with depressive (but not anxiety symptoms) in girls. Most studies exploring these associations in a community sample of adolescents have only considered hair cortisol concentrations and have reported mixed findings. For example, a study found no link between hair cortisol concentrations and depressive symptoms in pre-to-early adolescent girls (10–12 years old, [32]), while another study found a positive association between these two variables in a sample of adolescent boys and girls, although sex differences were not tested (12–21 years old, [33]). The broad age range used in some studies could explain discrepancies reported in the literature. Indeed, including participants with a wide age range and spanning different pubertal stages can introduce biases or mask results due to the interaction between sex and stress hormones. For instance, some studies have shown a stronger link between cortisol and depressive symptoms in adolescents with advanced to completed pubertal development relative to adolescents who have not started puberty or are at early stages [51,52]. The present sample comprises 14–15-year-old adolescent girls and boys in mid-puberty stage, which can possibly explain the lack of significant associations between hair cortisol and depressive symptoms, a link that could emerge when puberty development is more advanced or completed. Although the moderating effect of puberty was not tested due to lack of statistical power, this variable was controlled for in the analysis. This highlights the need for future studies to investigate how the relationship between glucocorticoids, internalizing symptoms, and sex may be moderated by puberty. Another possible explanation could be that studies in this domain use statistical cut-offs to create ‘’high” and ‘’low’’ cortisol concentrations groups [33,34]. Using continuous scores provides a more nuanced understanding of the relationship than using cut-offs and adds power to the statistical models. Furthermore, no associations were found between hair glucocorticoids and anxiety symptoms in either girls or boys. Very few studies have explored this link in a community sample and those that have, reported mixed results, with studies finding either a negative association between hair cortisol and anxiety symptoms only in girls or no association regardless of sex [32,35]. A possible explanation for our null finding could be the lack of variability in the anxiety symptoms scores, since most of the scores were clustered around the mean. Although girls reported significantly more anxiety symptoms than boys, both sexes still had relatively low scores.

Our study shows a significant association between hair cortisone concentrations and depressive symptoms, but only in girls (Fig. 1). This finding highlights the importance of considering both hormones when exploring internalizing symptoms. To date, there has been a notable lack of research examining hair cortisone concentrations, particularly among adolescents from community samples. Those few studies that explored cortisone concentrations have done so in relation to stress related variables (i.e., physiological symptoms, coping styles, emotions after a stressful event) in children and found significant positive associations, with higher cortisone concentrations linked to higher stress related symptoms [27,38]. More specifically, Molenaar and colleagues [27] found a significant positive relationship between hair cortisone concentrations and exposure to prenatal maternal stress in children, and this link was not found with cortisol concentrations. Bearing in mind the current limited literature on hair cortisone concentrations, one possible explanation could be that HPA axis dysregulation may initially manifest through changes in cortisone concentrations (early marker of mental health dysregulations) before having an impact on cortisol concentrations (more indicative of significant hormonal dysregulations) which have been linked more frequently to mental health dysregulation [20,26]. This would be consistent with our finding that cortisol concentration was not associated with depressive symptoms in boys or girls since we investigated those links in a community sample, where symptom severity is relatively low. Indeed, several studies on individuals with depressive disorders have explored the role of 11β-HSD type 2 to better understand hair cortisol concentration differences among those with depression compared to healthy controls. Findings showed a downregulation of this enzyme, leading to greater cortisol concentrations in depressed individuals [13,15,30]. While there was no measure of this enzyme in the present study, one hypothesis is that 11β-HSD type 2 could compensate as a way of regulating hormones, degrading more cortisol into cortisone. Similarly, a few studies with clinical populations have shown significant differences on the cortisol/cortisone ratio (biomarker representing 11β-HSD type 1 and 2 activity) between depressed and non-depressed individuals [29,30,53]. Hence, the non-significant association between the cortisol/cortisone ratio and depressive symptoms in a community sample is not surprising. More studies on hair cortisone are needed to confirm its role in the development of depressive symptoms. Studies show significant changes and impairments in brain regions where glucocorticoid receptors are predominant, such as the prefrontal cortex and the limbic system, in adolescents and adults in clinical sample [54]. Our results suggest that it may be particularly important to focus on cortisol levels early in development to ensure the maintenance of physiological homeostasis, as an early imbalance could disrupt brain physiology. Nevertheless, the present study highlights that hair cortisone concentrations may yield important and complementary information on HPA axis functioning and its associations with internalizing symptoms.

Importantly, results differed between boys and girls. Indeed, no significant associations were found between hair cortisol and cortisone concentrations and their ratio in relation to depression or anxiety symptoms in boys, whereas among girls, higher hair cortisone concentration was positively associated with depressive symptoms. These sex-dependent results add to the literature since studies to date have mainly explored this link only in girls [38,55] or have not disaggregated analyses based on sex [33,37]. One possible explanation for the differences in results between sexes could be that adolescent girls showed significantly higher internalizing symptoms than adolescent boys, which could indicate a greater level of burden for girls and explain their association with cumulative hair cortisone concentrations. Another explanation might be that adolescent boys in our sample are significantly less advanced (early puberty stage) in their puberty development than adolescent girls (mid puberty stage), even though we controlled for puberty status. We might see similar associations in boys if they were more advanced on the puberty scale. Indeed, research shows a possible interaction between the hypothalamic-pituitary-gonadal (HPG) axis and the HPA axis starting at puberty [51]. Also, sex hormones (i.e., testosterone, estradiol) seem to play a role in the regulation of internalizing and externalizing symptoms [56]. Exploring the role of these hormones in future research could help to clarify links between stress hormones and internalizing symptoms in adolescents. Taking together, results highlight the importance of considering sex differences and pubertal timing in analyses, such that associations between hair glucocorticoids and internalizing symptoms may be different in adolescent girls and boys. More studies are needed to identify if these associations might differ based on pubertal status.

This study has many strengths. First, it is one of few studies that has explored these associations in a community sample of adolescents. We also employed a cumulative measure of glucocorticoids, which is less sensitive to daily fluctuation and represents the functioning of the HPA axis over a longer period of time. Further, we explored not only hair cortisol concentrations, the most studied stress hormone, but also hair cortisone concentrations and their ratio. Hence, by exploring cortisone, we were able to gain insights that could indicate early signs of hormonal dysregulation, information that could have been missed by exploring cortisol alone. Another important strength is that analyses included a more careful examination of sex differences in associations between glucocorticoids and internalizing symptoms. This is important since internalizing symptoms and disorders affect a larger proportion of girls and women than boys and men. This study is not without limits. The cross-sectional nature of this study makes it impossible to study directionality of associations. The data were drawn from a larger study where certain relevant variables, such as gender identity, were not available. Also, the question about sex at birth (‘’are you a boy or a girl?’’) was not clearly defined and may be interpreted as referring to gender rather than biological sex. To reduce the impact of this limit, we verified that the sex reported by the adolescent in the present study concorded with the sex report by their parents at age two and age eight years. No discrepancies were found. Given the potential differences in physiological reactivity and emotional expression between genders, future research should more carefully examine sex and gender to better understand their roles in links between glucocorticoids and internalizing symptoms [57]. The lack of data on contraceptive use represents another limit as this factor could influence hormonal regulation and stress sensitivity [14]. Although we controlled for pubertal status, we did not use the other measures from the LC-MS extraction about sex hormone concentrations (i.e. testosterone, progesterone, estradiol), which could interact with glucocorticoids. Some adolescents in the sample were taking medication, and although no differences were found in hair glucocorticoid levels between medicated and non-medicated participants, we cannot fully rule out potential effects of medication on these measures. Also, given that adolescent diagnoses and medication use were self-reported by parents, we were unable to validate this information. It is also possible that some adolescents might be taking medication without their parents' knowledge, given that in Québec, the legal age to consult a healthcare professional without parental consent is 14 years old. This could contribute to underreporting of medication use when relying solely on parent-reported data. Future studies could incorporate clinical assessments to confirm these reports. Finally, despite having enough participants for statistical analyses, the relatively small sample size and the homogeneity of sociodemographic characteristics in our sample limits the generalizability of our findings. It would have also been relevant to acquire information on perceived stress and coping mechanisms since this could enable us to explore other mechanisms (such as stress perception and appraisal) that could modulate these associations and to explore how these associations would evolve over time (i.e., changes in symptoms or in hair glucocorticoid concentrations).

In conclusion, this study provides valuable insights into the associations between hair glucocorticoids and internalizing symptoms in adolescents from a community sample, a population that remains underexplored. Our findings also provide a more nuanced understanding of the sex differences observed in the prevalence of internalizing symptoms starting in adolescence. Further research is needed in community samples to address internalizing symptoms more effectively in diverse populations. Knowledge on associations between hair glucocorticoids and internalizing symptoms could help inform about potential hormonal dysregulation as an indicator for early interventions with adolescents at risk of developing internalizing disorders.

## CRediT authorship contribution statement

**Yasmine Zerroug:** Writing – review & editing, Writing – original draft, Visualization, Methodology, Investigation, Formal analysis, Conceptualization. **Marie-France Marin:** Writing – review & editing, Validation, Supervision, Investigation, Formal analysis, Conceptualization. **Mara Brendgen:** Writing – review & editing, Funding acquisition, Data curation, Conceptualization. **Miriam Beauchamp:** Writing – review & editing, Funding acquisition, Data curation, Conceptualization. **Jean R. Séguin:** Writing – review & editing, Funding acquisition, Data curation, Conceptualization. **Sylvana M. Côté:** Writing – review & editing, Funding acquisition, Data curation, Conceptualization. **Catherine M. Herba:** Writing – review & editing, Validation, Supervision, Investigation, Funding acquisition, Conceptualization.

## Funding

The Canadian Institutes of Health Research (CIHR) (MOP- 79420, IHD-107532, and POH-120254), the Canadian Council for Learning and the 10.13039/100021638Social Sciences and Humanities Research Council (SSHRC) (435-2016-1259) supported this study. Zerroug holds a bursary from the Fonds de recherche de la Santé du Québec (10.13039/501100000156FRQS), Marin holds funding from her Canada Research Chair in Hormonal Modulation of Cognitive and Emotional Functions and Herba received a salary award from 10.13039/501100000156FRQS and holds a strategic chair in Perinatal mental health and family well-being from the Université du Québec à Montréal (10.13039/100009452UQAM).

## Declaration of competing interest

The authors report no conflict of interest.

## References

[1] Blomqvist I., Henje Blom E., Hägglöf B., Hammarström A. (2019). Increase of internalized mental health symptoms among adolescents during the last three decades. Eur. J. Publ. Health.

[2] Keyes K.M., Platt J.M. (2024). Annual Research Review: sex, gender, and internalizing conditions among adolescents in the 21st century – trends, causes, consequences. Child Psychology Psychiatry.

[3] Lee F.S., Heimer H., Giedd J.N., Lein E.S., Šestan N., Weinberger D.R., Casey B.J. (2014). Adolescent mental health—opportunity and obligation. Science.

[4] Melton T.H., Croarkin P.E., Strawn J.R., Mcclintock S.M. (2016). Comorbid anxiety and depressive symptoms in children and adolescents: a systematic review and analysis. J. Psychiatr. Pract..

[5] Shorey S., Ng E.D., Wong C.H. (2022). Global prevalence of depression and elevated depressive symptoms among adolescents: a systematic review and meta-analysis. Br. J. Clin. Psychol..

[6] Alloy L.B., Hamilton J.L., Hamlat E.J., Abramson L.Y. (2016). Pubertal development, emotion regulatory styles, and the emergence of sex differences in internalizing disorders and symptoms in adolescence. Clin. Psychol. Sci..

[7] Kuehner C. (2017). Why is depression more common among women than among men?. Lancet Psychiatry.

[8] Morken I.S., Viddal K.R., Von Soest T., Wichstrøm L. (2023). Explaining the female preponderance in adolescent depression—a four-wave cohort study. Res Child Adolesc Psychopathol.

[9] Salk R.H., Hyde J.S., Abramson L.Y. (2017). Gender differences in depression in representative national samples: meta-analyses of diagnoses and symptoms. Psychol. Bull..

[10] Dallman M.F. (2005). Fast glucocorticoid actions on brain: back to the future. Front. Neuroendocrinol..

[11] Gerritsen L., Staufenbiel S.M., Penninx B.W., van Hemert A.M., Noppe G., de Rijke Y.B., van Rossum E.F. (2019). Long-term glucocorticoid levels measured in hair in patients with depressive and anxiety disorders. Psychoneuroendocrinology.

[12] Greff M.J., Levine J.M., Abuzgaia A.M., Elzagallaai A.A., Rieder M.J., van Uum S.H. (2019). Hair cortisol analysis: an update on methodological considerations and clinical applications. Clin. Biochem..

[13] Koss K.J., Gunnar M.R. (2018). Annual research review: early adversity, the hypothalamic–pituitary–adrenocortical axis, and child psychopathology. JCPP (J. Child Psychol. Psychiatry).

[14] Staufenbiel S.M., Penninx B.W., de Rijke Y.B., van den Akker E.L., van Rossum E.F. (2015). Determinants of hair cortisol and hair cortisone concentrations in adults. Psychoneuroendocrinology.

[15] Rippe R.C., Noppe G., Windhorst D.A., Tiemeier H., van Rossum E.F., Jaddoe V.W., Verhulst F.C., Bakermans-Kranenburg M.J., van IJzendoorn M.H., van den Akker E.L. (2016). Splitting hair for cortisol? Associations of socio-economic status, ethnicity, hair color, gender and other child characteristics with hair cortisol and cortisone. Psychoneuroendocrinology.

[16] Herman J.P. (2022). The neuroendocrinology of stress: glucocorticoid signaling mechanisms. Psychoneuroendocrinology.

[17] Sisk L.M., Gee D.G. (2022). Stress and adolescence: vulnerability and opportunity during a sensitive window of development. Curr. Opin. Psychol..

[18] Dettenborn L., Muhtz C., Skoluda N., Stalder T., Steudte S., Hinkelmann K., Kirschbaum C., Otte C. (2012). Introducing a novel method to assess cumulative steroid concentrations: increased hair cortisol concentrations over 6 months in medicated patients with depression. Stress.

[19] Kirschbaum C., Tietze A., Skoluda N., Dettenborn L. (2009). Hair as a retrospective calendar of cortisol production—increased cortisol incorporation into hair in the third trimester of pregnancy. Psychoneuroendocrinology.

[20] Meyer J.S., Novak M.A. (2021). Assessment of prenatal stress‐related cortisol exposure: focus on cortisol accumulation in hair and nails. Dev. Psychobiol..

[21] Guerry J.D., Hastings P.D. (2011). In search of HPA axis dysregulation in child and adolescent depression. Clin. Child Fam. Psychol. Rev..

[22] Lopez-Duran N.L., Kovacs M., George C.J. (2009). Hypothalamic–pituitary–adrenal axis dysregulation in depressed children and adolescents: a meta-analysis. Psychoneuroendocrinology.

[23] Vives A.H., De Angel V., Papadopoulos A., Strawbridge R., Wise T., Young A.H., Arnone D., Cleare A.J. (2015). The relationship between cortisol, stress and psychiatric illness: new insights using hair analysis. J. Psychiatr. Res..

[24] Steudte-Schmiedgen S., Wichmann S., Stalder T., Hilbert K., Muehlhan M., Lueken U., Beesdo-Baum K. (2017). Hair cortisol concentrations and cortisol stress reactivity in generalized anxiety disorder, major depression and their comorbidity. J. Psychiatr. Res..

[25] Psarraki E.E., Kokka I., Bacopoulou F., Chrousos G.P., Artemiadis A., Darviri C. (2021). Is there a relation between major depression and hair cortisol? A systematic review and meta-analysis. Psychoneuroendocrinology.

[26] Jiang Z., Dong L., Zhang Y., Mao H., Luo F., Song M. (2025). Cortisol levels and depression suicide risk: a combined exploration of meta-analysis and case-control study. Front. Psychiatr..

[27] Molenaar N.M., Tiemeier H., van Rossum E.F., Hillegers M.H.J., Bockting C.L.H., Hoogendijk W.J.G., Van Den Akker E.L., Lambregtse-van den Berg M.P., El Marroun H. (2019). Prenatal maternal psychopathology and stress and offspring HPA axis function at 6 years. Psychoneuroendocrinology.

[28] Stalder T., Kirschbaum C., Alexander N., Bornstein S.R., Gao W., Miller R., Stark S., Bosch J.A., Fischer J.E. (2013). Cortisol in hair and the metabolic syndrome. J. Clin. Endocrinol. Metabol..

[29] Zerroug Y., Marin M.-F., Porter-Vignola É., Garel P., Herba M.C. (2025). Differences in hair cortisol to cortisone ratio between depressed and non-depressed adolescent women. Stress.

[30] Zhang J., Li J., Xu Y., Yang J., Chen Z., Deng H. (2013). Characteristics of novel hair-based biomarker for the activity assessment of 11β-hydroxysteroid dehydrogenase. Clin. Chim. Acta.

[31] Rao U., Chen L.-A. (2022). Characteristics, correlates, and outcomes of childhood and adolescent depressive disorders. Dialogues Clin. Neurosci..

[32] Lu Q., Pan F., Ren L., Xiao J., Tao F. (2018). Sex differences in the association between internalizing symptoms and hair cortisol level among 10-12 year-old adolescents in China. PLoS One.

[33] Rietschel L., Streit F., Zhu G., McAloney K., Kirschbaum C., Frank J., Hansell N.K., Wright M.J., McGrath J.J., Witt S.H. (2016). Hair cortisol and its association with psychological risk factors for psychiatric disorders: a pilot study in adolescent twins. Twin Res. Hum. Genet..

[34] Gerber M., Kalak N., Elliot C., Holsboer-Trachsler E., Pühse U., Brand S. (2013). Both hair cortisol levels and perceived stress predict increased symptoms of depression: an exploratory study in young adults. Neuropsychobiology.

[35] Gray N.A., Dhana A., Van Der Vyver L., Van Wyk J., Khumalo N.P., Stein D.J. (2018). Determinants of hair cortisol concentration in children: a systematic review. Psychoneuroendocrinology.

[36] Stalder T., Steudte-Schmiedgen S., Alexander N., Klucken T., Vater A., Wichmann S., Kirschbaum C., Miller R. (2017). Stress-related and basic determinants of hair cortisol in humans: a meta-analysis. Psychoneuroendocrinology.

[37] Ford J.L., Boch S.J., Browning C.R. (2019). Hair cortisol and depressive symptoms in youth: an investigation of curvilinear relationships. Psychoneuroendocrinology.

[38] Vanaelst B., Michels N., De Vriendt T., Huybrechts I., Vyncke K., Sioen I., Bammann K., Rivet N., Raul J.-S., Molnar D., De Henauw S. (2013). Cortisone in hair of elementary school girls and its relationship with childhood stress. Eur. J. Pediatr..

[39] Charrois J., Côté S.M., Japel C., Séguin J.R., Paquin S., Tremblay R.E., Herba C.M. (2017). Child‐care quality moderates the association between maternal depression and children's behavioural outcome. Child Psychology Psychiatry.

[40] Côté S.M., Mongeau C., Japel C., Xu Q., Séguin J.R., Tremblay R.E. (2013). Child care quality and cognitive development: trajectories leading to better preacademic skills. Child Dev..

[41] Gingras M.-P., Brendgen M., Beauchamp M.H., Séguin J.R., Tremblay R.E., Côté S.M., Herba C.M. (2023). Adolescents and social media: longitudinal links between types of use, problematic use and internalizing symptoms. Res Child Adolesc Psychopathol.

[42] Petersen A.C., Crockett L., Richards M., Boxer A. (1988). A self-report measure of pubertal status: reliability, validity, and initial norms. J. Youth Adolesc..

[43] Geoffroy M.-C., Boivin M., Arseneault L., Turecki G., Vitaro F., Brendgen M., Renaud J., Séguin J.R., Tremblay R.E., Côté S.M. (2016). Associations between peer victimization and suicidal ideation and suicide attempt during adolescence: results from a prospective population-based birth cohort. J. Am. Acad. Child Adolesc. Psychiatr..

[44] Kovacs M. (2011).

[45] Reynolds C., Richmond B.O. (1985). Revised children's manifest anxiety scale. Psychol. Assess..

[46] Enge S., Fleischhauer M., Hadj-Abo A., Butt F., Kirschbaum C., Schmidt K., Miller R. (2020). Comparison of hair cortisol concentrations between self-and professionally-collected hair samples and the role of five-factor personality traits as potential moderators. Psychoneuroendocrinology.

[47] Ouellet-Morin I., Laurin M., Robitaille M.-P., Brendgen M., Lupien S.J., Boivin M., Vitaro F. (2016). Validation of an adapted procedure to collect hair for cortisol determination in adolescents. Psychoneuroendocrinology.

[48] Gao W., Stalder T., Foley P., Rauh M., Deng H., Kirschbaum C. (2013). Quantitative analysis of steroid hormones in human hair using a column-switching LC–APCI–MS/MS assay. J. Chromatogr. B.

[49] Schindler-Gmelch L., Capito K., Steudte-Schmiedgen S., Kirschbaum C., Berking M. (2024). Hair cortisol research in posttraumatic stress disorder-10 Years of insights and open questions. A systematic review. Curr. Neuropharmacol..

[50] Cohen J. (1988).

[51] Marceau K., Ruttle P.L., Shirtcliff E.A., Essex M.J., Susman E.J. (2015). Developmental and contextual considerations for adrenal and gonadal hormone functioning during adolescence: implications for adolescent mental health. Dev. Psychobiol..

[52] Reynolds R.M., Hii H.L., Pennell C.E., McKeague I.W., de Kloet E.R., Lye S., Stanley F.J., Mattes E., Foster J.K. (2013). Analysis of baseline hypothalamic-pituitary-adrenal activity in late adolescence reveals gender specific sensitivity of the stress axis. Psychoneuroendocrinology.

[53] Römer B., Lewicka S., Kopf D., Lederbogen F., Hamann B., Gilles M., Schilling C., Onken V., Frankhauser P., Deuschle M. (2009). Cortisol metabolism in depressed patients and healthy controls. Neuroendocrinology.

[54] Trifu S.C., Trifu A.C., Aluaş E., Tătaru M.A., Costea R.V. (2020). Brain changes in depression. Rom. J. Morphol. Embryol..

[55] Sandstrom A., Daoust A.R., Russell E., Koren G., Hayden E.P. (2021). Hair cortisol concentrations predict change in girls' depressive symptoms. Eur. J. Dev. Psychol..

[56] Chronister B.N., Gonzalez E., Lopez-Paredes D., Suarez-Torres J., Gahagan S., Martinez D., Barros J., Jacobs Jr D.R., Checkoway H., Suarez-Lopez J.R. (2021). Testosterone, estradiol, DHEA and cortisol in relation to anxiety and depression scores in adolescents. J. Affect. Disord..

[57] Juster R.-P. (2019). Sex × gender and sexual orientation in relation to stress hormones and allostatic load. Gender and the Genome.

